# A complex of novel protease inhibitor, ovostatin homolog, with its cognate proteases in immature mice uterine luminal fluid

**DOI:** 10.1038/s41598-019-41426-4

**Published:** 2019-03-21

**Authors:** Hsien-Lu Huang, Szu-Chin Li, Jin-Fong Wu

**Affiliations:** 1 Department of Nutrition and Health Science, Fooyin University, Kaohsiung City, 83102 Taiwan; 2 Department of Biomedical Sciences, National Chung Cheng University, Chiayi, 62102 Taiwan; 3 School of Medicine, Tzu Chi University, Hualien, 97004 Taiwan; 4 Division of Hematology-Oncology, Department of Internal medicine, Dalin Tzu Chi Hospital, Buddhist Tzu Chi Medical Foundation, Chiayi, 62247 Taiwan

## Abstract

A predominant gelatinolytic enzyme with approximately 26 kDa was observed in gelatin zymogram of immature mice uterine luminal fluid (ULF). Size exclusion analysis revealed that the native size of this enzyme was close to that of human α_2_-macroglobulin (α_2_-MG), a 725 kDa protein. This large protease was isolated by a series of chromatographic steps on the Sephacryl S-400 and DEAE-Sepharose columns. The results from gelatin zymography and SDS-PAGE analysis supported that this large protease consists of gelatinolytic enzyme and a 360 kDa protein. Through tandem mass spectrometry analysis followed by MASCOT database search, the 360 kDa protein was identified as ovostatin homolog (accession: NP_001001179.2) assigned as a homolog of chicken ovostatin, a protease inhibitor. The co-fractionation analysis by gel filtration and mouse ovostatin homolog (mOH) co-immunoprecipitation experiments demonstrated that the mOH formed a complex with three gelatinolytic enzymes in immature mice ULF. Substrate zymography analysis revealed that the mOH-associated gelatinolytic enzymes were suitable to digest type I collagen rather than type IV collagen. In addition, the refolded mOH-associated 26 kDa gelatinolytic enzyme displayed the type I collagen-digesting activity in the assay, but the other two enzymes did not have this function. RT-PCR analysis showed that mOH gene was abundantly expressed in brain, spinal cord, lung, uterus, and in 17-day embryo. Taken together, our data suggest that mOH/cognate protease system may play a potential role in regulation of tissue remodeling and fetal development.

## Introduction

In mice, the uterine endometrium undergoes extensive remodeling during estrous cycle. This involves endometrial cell proliferation, differentiation, and apoptosis in accompany with extracellular matrix (ECM) remodeling under the influence of ovarian steroids^1,2^. ECM proteins can be degraded by a variety of enzymes. Among them, collagen-degrading enzymes play an important role because collagens, the main protein components of ECM, provide tissues with their strength and structure. Many collagen-degrading enzymes also strongly degrade the denatured collagen (gelatin) and correspond to gelatinolytic enzymes, which can be detected by gelatin zymography^3,4^. Many studies had been carried out to analyze the expression of gelatinolytic enzymes in the mammalian endometrium^5–8^. Several mechanisms exist to regulate the activities of these gelatinolytic enzymes during the tissue remodeling^9^. Alternatively, the mechanism involved in their cognate inhibitors plays important physiological role in these events^10,11^. The cognate inhibitors of gelatinolytic enzymes including the tissue inhibitors of metalloprotease (TIMPs) and plasminogen activator (PA) inhibitors have been implicated to participate in several uterine processes such as ovarian cycle^12,13^, implantation^14–16^, and placentation^17,18^. Although all these events share the general feature of tissue remodeling, the molecular bases of these events have not been thoroughly worked out. Several lines of evidence had exhibited to support the contention that MMPs were key players at endometrial breakdown and repair^19,20^. However, this argument was not supported by mouse model studies^21^, raising the possibility that the unidentified collagen-degrading enzymes may exist in mouse endometrium to share these processes. To test the possibility, the novel gelatinolytic enzymes in mouse endometrium need to be isolated and characterized. However, the isolation of these minor proteins from mouse total uterine proteins was very difficult^22^.

In mouse endometrium, the columnar epithelium forms the central lumen and glands that pass through the supporting stroma. Under the estrogen surge, mouse endometrial cells intensely proliferate. At the same time, the proteins secreted from proliferative endometrium move into lumen and accumulate in luminal fluid^23^. The soluble gelatinolytic enzymes secreted from changeable endometrium may serve as the ECM destructor and exist in accessible body fluid. In addition, the presence of cognate inhibitors in the efflux may reflect the underlying mechanism for the regulation of ECM remodeling. The quantity of uterine luminal fluid (ULF) mainly appears in mouse pro-estrous and estrous stages^24,25^. Nevertheless, the collection of a great deal of mature mouse ULF from the specific stages was difficult. It is well-known that the stimulation of immature mice by estrogen or its analogues results in endometrial growth accompanying with a remarkable increase in ULF^26–28^, providing a good experimental system to isolate or purify the estrogen-stimulated gelatinolytic enzymes for functional characterization and antibody production.

In the present study, we identified and characterized a novel protein, mouse ovostatin homolog (mOH), in immature mice ULF, demonstrating that the mOH was a serine proteases inhibitor specific for a 26 kDa type I collagen-degrading enzyme. Taken together, our findings provided the basis for unveiling and understanding the function of mOH in ECM remodeling.

## Results

### The gelatinolytic enzyme complex is one of the components in ULF

**Figure 1:**
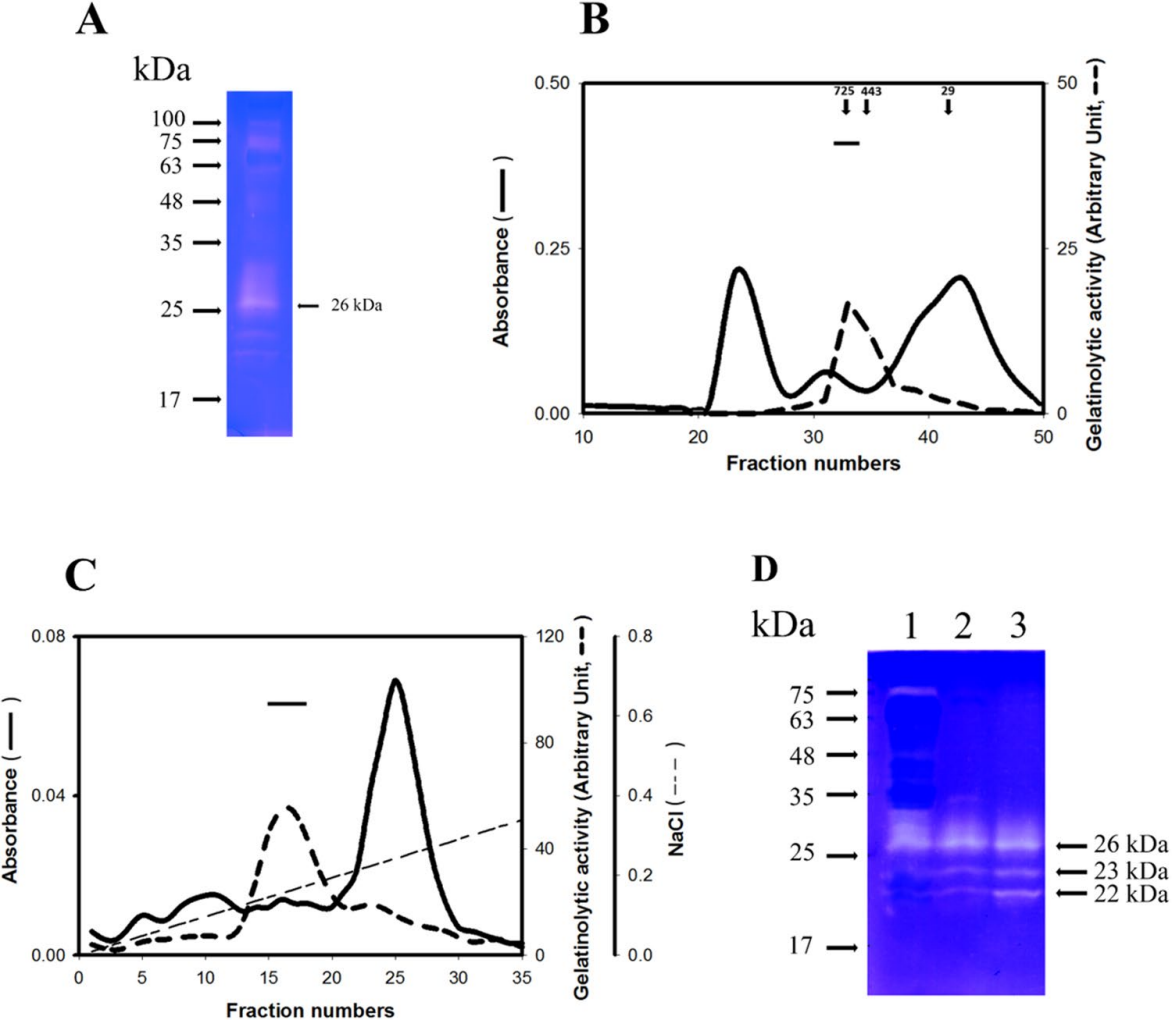
Purification of the ULF gelatinolytic enzyme. (**A**) Gelatin zymogram of immature mouse ULF. The ULF (6 μl) was analyzed by gelatin zymography. (**B**) Elution profile of ULF gelatinolytic enzyme carried out by gel filtration chromatography. Soluble ULF (1 ml) was subjected to gel filtration chromatography on a Sephacryl S-400 column. The elution was monitored by UV spectrophotometry with absorbance at 280 nm (—). The gelatinolytic activity of 26 kDa enzyme in each indicated fraction was quantitatively detected (---). Arrows indicate the protein markers eluted from the same column. The protein markers are human α_2_MG (725 kDa), apoferritin (443 kDa) and carbonic anhydrase (29 kDa). The horizontal bar represents the pooled fractions for further purification by the DEAE-Sepharose chromatography. (**C**) Elution profile of DEAE-Sepharose chromatography. The pooled fractions are marked by a horizontal bar. (**D**) Gelatin zymographic assay. Lane 1: immature mouse ULF (6 μl). Lane 2: the pooled fractions (0.3 ml) from gel filtration. Line 3: the pooled fractions (0.25 ml) from DEAE-Sepharose chromatography.

Stimulation of immature mice by estrogen caused its endometrial proliferation in combination with a remarkable secretion of ULF. To ask whether ECM-degrading enzymes are secreted by endometrial cells for tissue remodeling, gelatin zymography of immature mice ULF was performed (Fig. 1A). Several gelatinolytic enzymes in the soluble ULF were shown in the gelatin zymogram (Fig. 1A). Especially, a prominent negative staining band with apparently molecular weight of 26 kDa was observed, suggesting that it is different to that of plasminogen activators including tissue PA or urokinase^29^.

To isolate the 26 kDa gelatinolytic enzyme from ULF, the soluble ULF was chromatographed on a Sephacryl-S-400 column. The elution profile of gel filtration was shown in Fig. 1B, revealing that the native size of 26 kDa gelatinolytic enzyme was close to 725 kDa. This consequence suggested that the 26 kDa protease may be associated with its inhibitor or substrate. To isolate this protease complex, the fractions pooled from gel filtration were re-chromatographed on a DEAE-Sepharose column. The elution profile was displayed in Fig. 1C. The peak of gelatinolytic enzyme complex is appeared at approximately 0.15 M NaCl. Figure 1D also shows that two new species of gelatinolytic enzymes (23 and 22 kDa) were co-eluted during purification.

### mOH is one of the components in the gelatinolytic enzyme complex

**Figure 2:**
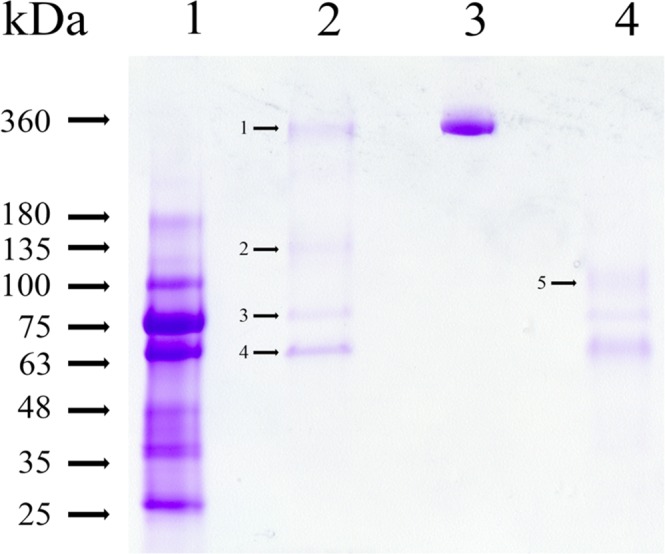
SDS-PAGE analysis of the gelatinolytic enzyme complex isolated from immature mice ULF. An aliquot was separated by a 4–15% gradient acrylamide gel with 0.1% SDS. Lane 1: ULF (5 μl) treated with 2-mercaptoethanol. Lane 2: the gelatinolytic enzyme complex (3 μg) without 2-mercaptoethanol treatment. Lane 3: human α_2_MG dimer (360 kDa, 3 μg). Lane 4: the gelatinolytic enzyme complex (3 μg) with 2-mercaptoethanol treatment. After electrophoresis, the gel was stained with Coomassie Brilliant Blue and then destained by 10% acetic acid. Five bands including 360, 135, 88, 75 and 66 kDa were numbered and indicated by the arrow bar.

Previous studies have indicated that α_2_-MG acts as a protease inhibitor and forms a complex with proteases. The apparent molecular weight of α_2_-MG complex is close to 700 kDa^30–32^. Thus, we asked whether α_2_-MG is present in immature mice ULF. The purified gelatinolytic enzyme complex and human α_2_-MG were analyzed by SDS-PAGE (Fig. 2). Under reducing conditions, many components of mice ULF were observed in the SDS-PAGE (Fig. 2, lane 1). The purified gelatinolytic enzyme complex consists of four major proteins displayed in non-reducing SDS-PAGE (Fig. 2, lane 2), whereas their apparent molecular weights are 360, 135, 75 and 66 kDa, respectively. No protein band at 26 kDa was observed in non-reducing polyacrylamide gel, suggesting that the low levels of gelatinolytic enzyme could not be detected by Coomassie Brilliant Blue staining. Of interest was the presence of a 360 kDa protein in gelatinolytic enzyme complex like that of human α_2_-MG dimer subunits (Fig. 2, lane 3). After reduction, both 360 and 135 kDa proteins were disappeared, while a faint band with the apparently molecular weight of 88 kDa was emerged in the gel (Fig. 2, lane 4), suggesting that the 88 kDa protein might be derived from the reduction of either 360 or 135 kDa protein.

To identify the proteins in the gelatinolytic enzymes complex, the bands from 1 to 5 marked in Fig. 2 were excised from the gel and subjected to in-gel digestion by trypsin, respectively. The molecular weights of released peptides were analyzed by LC-MS/MS and followed by data searching from the MASCOT database for protein identification. The result was shown in Table I. The 360 kDa protein in the gelatinolytic enzyme complex (Fig. 2, lane 2) was identified as mOH. The primary sequence of mOH is highly homologous to that of chicken ovostatin^33^. In addition, the primary sequence of ~88 kDa protein (Fig. 2, lane 4) showed a complete identity to that of mOH, suggesting that the 88 kDa protein is derived from the reduction of 360 kDa mOH. The mass spectrometry analysis also indicated that the complement component C3 (C3, 135 kDa), chloride channel calcium activated 3 (CLCA3, 75 kDa) and serum albumin (66 kDa) are present in the gelatinolytic enzymes complex (Table 1). However, the gelatinolytic enzyme complex does not contain mouse α_2_MG. The possibility of α_2_MG associated with the gelatinolytic enzymes in ULF was ruled out.

**Table 1:** List of five indicated proteins identified by Tandem Mass Spectrometry and MASCOT Search.

Indicated Band	Identified protein	Molecular Weight	Peptides matches	Sequence coverage	MASCOT score
1	Ovostatin homolog	360 kDa	21	15%	1120
2	Complement component C3	135 kDa	53	38%	1223
3	Chloride channel calcium activated 3	75 kDa	11	13%	625
4	Serum albumin	66 kDa	13	23%	762
5	Ovostatin homolog	88 kDa	13	9%	303

The five proteins viewed from Mascot Search Results were shown in Supplementary Fig. S2.1, S2.5.

### mOH, but not CLAC3, is associated with the gelatinolytic enzymes in ULF

**Figure 3:**
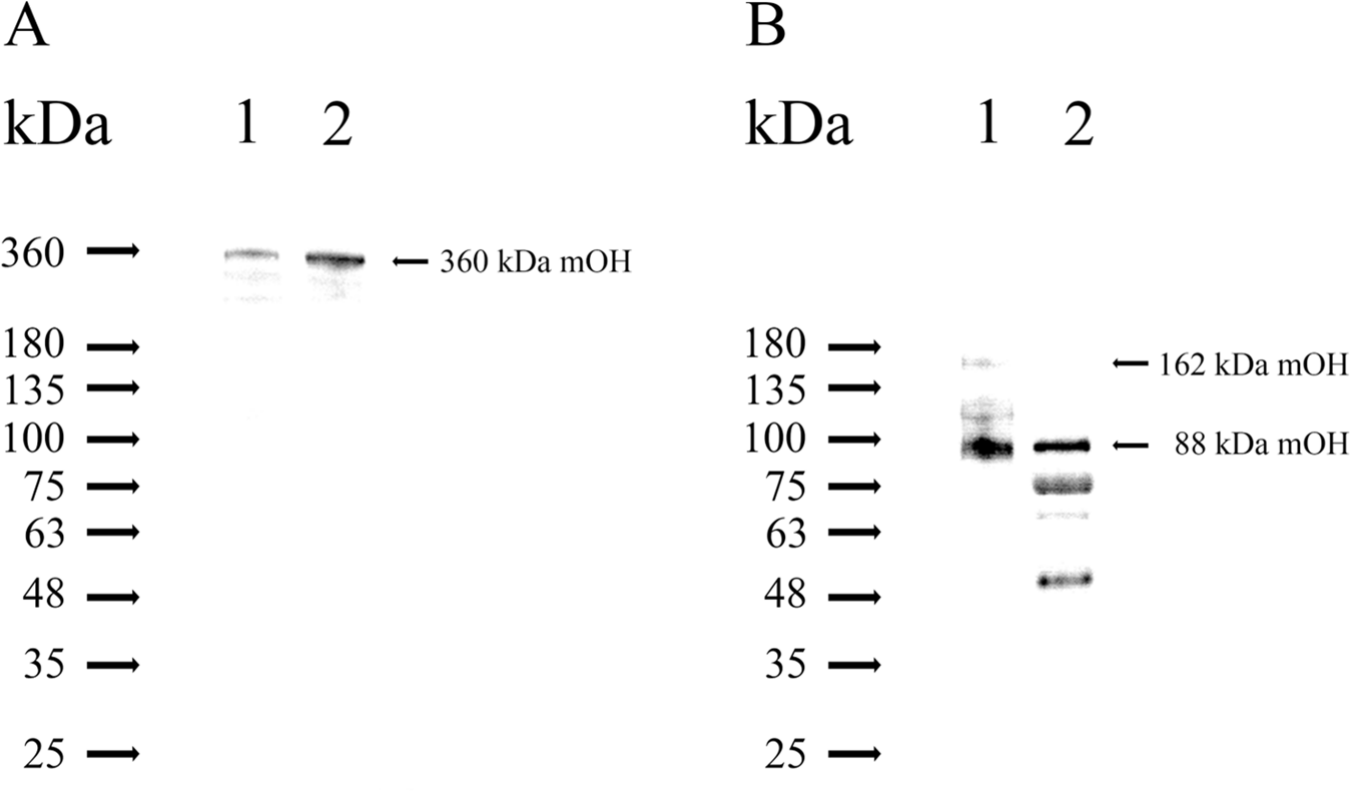
mOH is present in immature mouse ULF. (**A**) The immature mouse ULF and the gelatinolytic enzyme complex were separated by non-reducing SDS-PAGE and then analyzed by Western blotting, probed for mOH by POHA. The POHA was purified from rabbit anti-mOH sera. Lane 1: immature mouse ULF (5 μl). Lane 2: the gelatinolytic enzyme complex (0.5 μg) that was the resulting product after immature mouse ULF was purified by gel filtration and DEAE-Sepharose chromatography. (**B**) The immature mouse ULF and the gelatinolytic enzyme complex were separated by reducing SDS-PAGE and then analyzed by Western blotting, probed for reducing mOH by rabbit anti-GST-mOH[870–940] sera. Lane 1: immature mouse ULF (5 μl). Lane 2: the gelatinolytic enzyme complex (0.5 μg).

Both mOH and CLAC3 were co-eluted with gelatinolytic enzymes in the purification. Thus, we ask whether both proteins are associated with gelatinolytic enzymes in ULF. Firstly, we prepared the specific anti-mOH antibody. The gelatinolytic enzymes complex was used as the antigens to raise the antibody. Since the complex containing four proteins (Fig. 2, lane 2), the raised antibody can recognize all antigens. Thus, the raised antibody was subjected to gelatinolytic enzymes complex-conjugated beads. The elution solution was then absorbed with C3-, albumin- and CLCA3-conjugated beads. The remaining antibody named as purified mOH antibody (POHA) was subjected to recognition assay against the gelatinolytic enzyme complex and ULF by Western blotting. Figure 3A shows that POHA specifically recognizes the non-reduced mOH (360 kDa). Under reducing condition, GST-mOH[870–940] antibody recognizes the reduced mOH (88 kDa) in Western blotting assay (Fig. 3B). Two immunoreactive bands below 88 kDa mOH (Fig. 3B, lane 2) probably caused by proteolytic cleavage of 88 kDa during isolation procedure.

**Figure 4:**
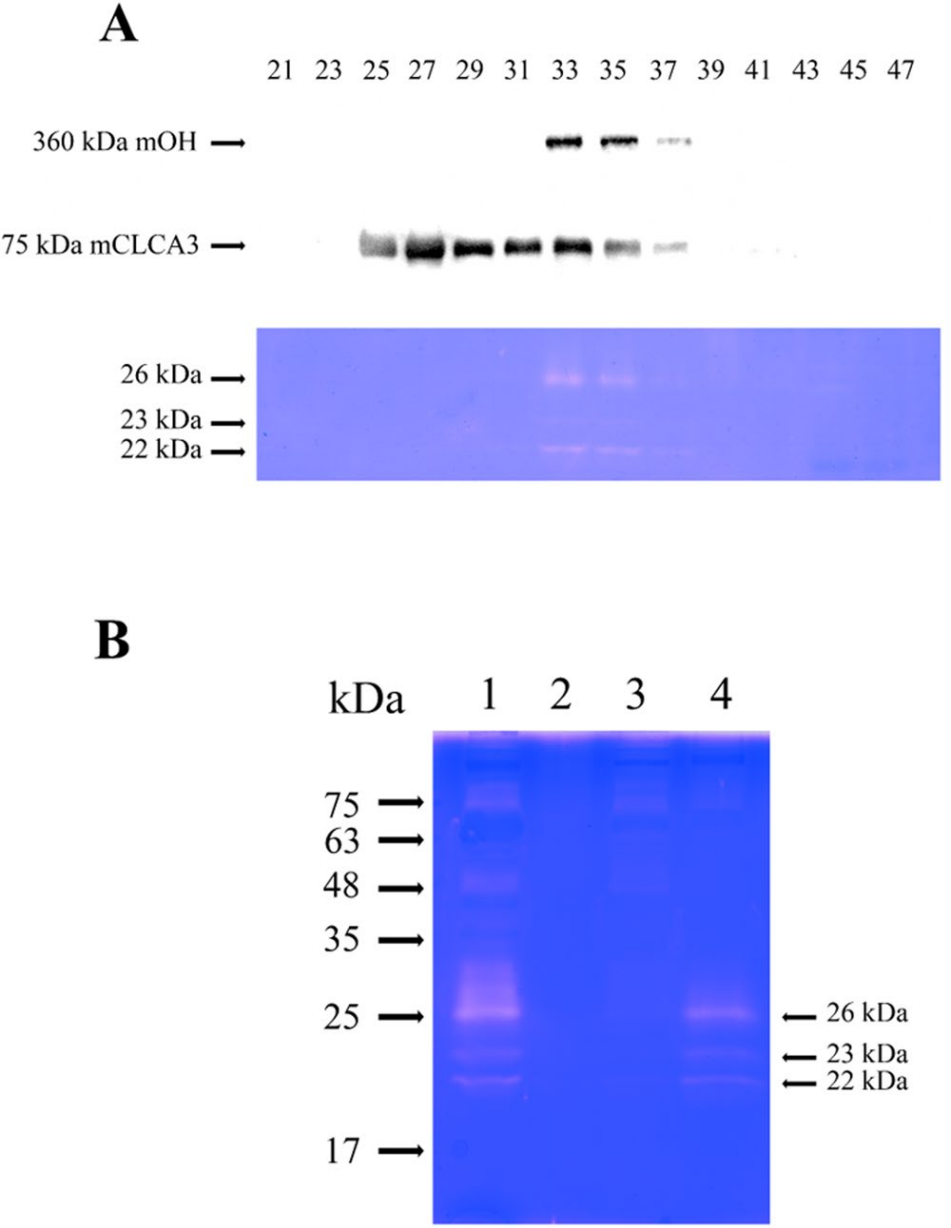
A complex of mOH with three gelatinolytic enzymes in immature mouse ULF. (**A**) Co-elution of mOH with gelatinolytic enzymes during purification of mOH by gel filtration. Each indicated fraction (0.3 ml) was separated by SDS-PAGE and then applied to immunoblot analysis for detection of mOH protein using POHA (upper panel) and for detection of mCLCA3 using anti-mCLCA3 antibody (middle panel), respectively. The identical samples were also subjected to gelatin zymography for detection of gelatinolytic enzymes (lower panel). The original immunoblots and scan of gelatin zymogram were displayed in Supplementary Fig. S3. (**B**) Co-immunoprecipitation of mOH with three gelatinolytic enzymes. Either POHA or preimmune antibody (PIA) was pre-immobilized on Protein A-Sepharose (60 μl), followed by incubation with immature mice ULF (30 μl). After centrifugation, the pellet was re-suspended in 30 μl of sample buffer. An aliquot of re-suspended pellet or supernatant was subjected to SDS-PAGE, followed by gelatin zymography for detection of gelatinolytic enzymes. Lane 1: PIA-treated ULF supernatant (6 μl). Lane 2: PIA-treated ULF pellet (6 μl). Lane 3: POHA-treated ULF supernatant (6 μl), Lane 4: POHA-treated ULF pellet (6 μl).

To verify whether both mOH and CLCA3 are associated with gelatinolytic enzymes, ULF was chromatographed on the Sephacryl S-400 column and its elution profile was analyzed by Western blotting using POHA and mCLCA3 antibody, respectively, and by gelatin zymography (Fig. 4A). Figure 4A shows that the mOH (360 kDa) was precisely co-eluted with gelatinolytic enzymes. Both were co-emerged between fraction 33 and 35, suggesting that mOH is associated with gelatinolytic enzymes. Nevertheless, these gelatinolytic enzymes were not detected in fractions containing CLCA3 (fraction 25–31). mOH co-eluted with gelatinolytic enzymes was also observed during purification by DEAE-Sepharose chromatography (Supplementary Fig. S1). A complex of mOH with gelatinolytic enzymes was also confirmed by co-immunoprecipitation from ULF by POHA (Fig. 4B).

### Characterization of the mOH-associated gelatinolytic enzyme activity

**Figure 5:**
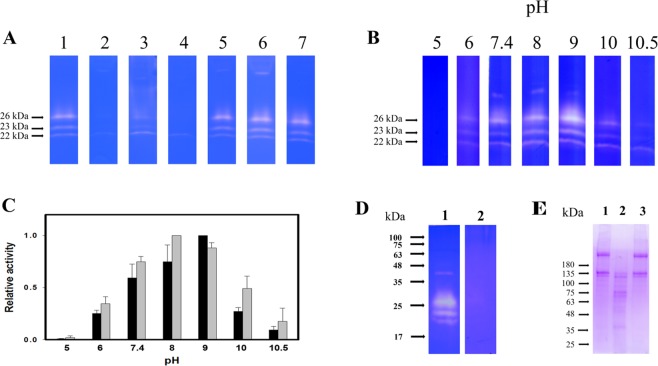
Characterization of mOH-associated gelatinolytic enzyme activity. (**A**) The effects of protease inhibitors on the activity of mOH-associated gelatinolytic enzymes. An aliquot (0.3 ml) isolated from gel filtration was subjected to SDS-PAGE, followed by gelatin zymographic assay. After electrophoresis, the gel was sliced, and each sliced gel was incubated without protease inhibitor (lane 1) or with different protease inhibitors. The protease inhibitors were bezamidine (2 mM, lane 2); chymostatin, (100 μM, lane 3); leupeptin (100 μM, lane 4); pepstatin (20 μM, lane 5); iodoacetamide (0.1 mM, lane 6); 1-10-phenanthroline (1 mM, lane 7). The original scans of gelatin zymograms were shown in Supplementary Fig. S5. (**B**) The effects of pH titration on the activity of mOH-associated gelatinolytic enzymes. An aliquot (0.3 ml) isolated from gel filtration was separated by SDS-PAGE, followed by gelatin zymographic assay in the presence of different pH buffers, respectively. The buffers include 0.1 M sodium citrate (pH 5.0 and 6.0), 0.1 M Tris buffers (from pH 7.4 and 8.0) and 0.1 M glycine (pH 9, 10 and 10.5). The original scans of gelatin zymograms were shown in Supplementary Fig. S6. (**C**) Quantitation of relative gelatinolytic activities averaged from three independent experiments. The highest activity in pH titration represent 100%. The optimal pH for the activity of 26 kDa (black bar) and 22 kDa gelatinolytic enzymes (gray bar) is 9 and 8, respectively. Means ± SD, n = 3. (**D**) The substrate specificity of mOH-associated gelatinolytic enzymes. An aliquot (2 μg) of gelatinolytic enzymes complex from purification was carried out for gelatin zymography in the presence of different substrates, type I collagen (lane 1) and type IV collagen (lane 2), respectively. (**E**) The type I collagen degraded by mOH-associated 26 kDa gelatinolytic enzyme. The free 26 and 23/22 kDa enzymes were incubated with type I collagen, respectively, as described in “Materials and Methods”. After overnight incubation, an aliquot (8 μL) of supernatant from each reaction mixture was applied to analysis by SDS-PAGE. Lane 1: incubation with blank gel. Lane 2: incubation with mOH-free 26 kDa enzyme. Lane 3: incubation with mOH-free 23/22 kDa enzymes.

The effects of protease inhibitors on the gelatinolytic activity of mOH-gelatinolytic enzymes were characterized. The gelatinolytic activity cannot be inhibited by pepstatin, iodoacetamide and 1–10-phenanthroline, suggesting that these gelatinolytic enzymes do not belong to aspartic proteases, cysteine proteases and metalloproteases (Fig. 5A). Benzamidine and leupeptin completely blocked the activity of 26 and 23 kDa gelatinolytic enzymes. In addition, chymostatin partially inhibits the activity of both 26 and 23 kDa gelatinolytic enzymes. However, the 22k kDa gelatinolytic enzyme shows more resistant to inhibition by benzamidine, leupeptin and chymostatin (Fig. 5A). These data also indicated that both 26 and 23 kDa gelatinolytic enzymes were distinct from the 22 kDa gelatinolytic enzyme.

The gelatinolytic activity of mOH-associated enzymes was also characterized by pH titration. Fig. 5B,C indicated that the optimal pH for digestion of gelatin is located between 8 and 9. None of the gelatinolytic enzymes shows the activity in acidic pH, suggesting that they were not lysosomal proteases released from endometrial cells. Furthermore, the substrate specificity of mOH-associated gelatinolytic enzymes was also tested. The type I collagen is the suitable substrate in the substrate zymography assay, but the type IV collagen resists to the digestion by mOH-associated gelatinolytic enzymes (Fig. 5D). In addition, the mOH-associated 26 kDa gelatinolytic enzyme extracted from the SDS-depleted gel displayed the type I collagen-digesting activity (Fig. 5E).

### Tissue distribution of mOH mRNA

**Figure 6:**
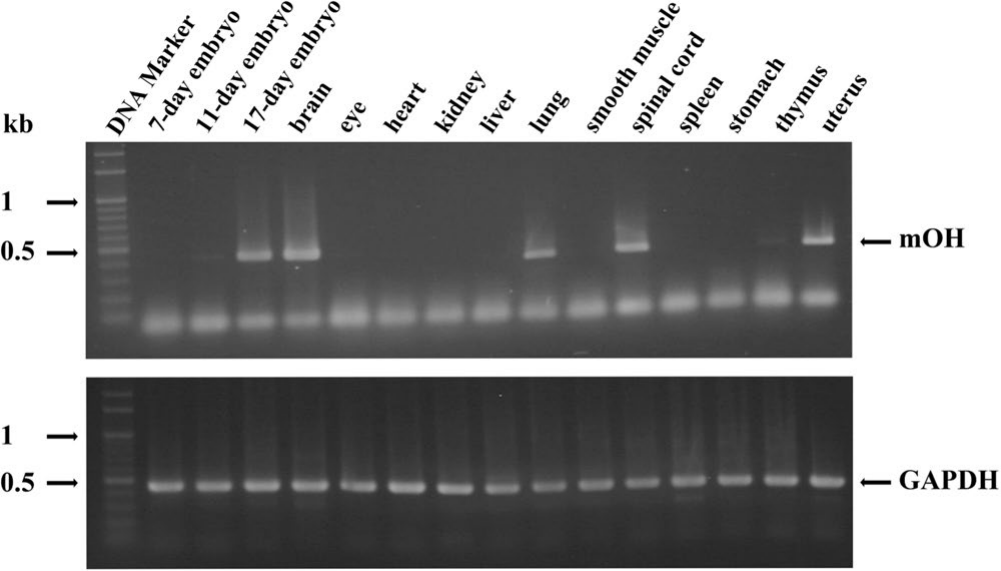
Tissue distribution of mOH mRNA. Total RNAs isolated from various adult mice tissues or from different stages of mice embryos were subjected to RT-PCR. The resulting products were separated in agarose gel and visualized by ethidium bromide staining under exposure of UV light. The RT-PCR product of GAPDH was served as the internal control. The original agarose gels were shown in Supplementary Fig. S7.

To determine the mOH gene expression in various mice tissues and embryo stages, we analyzed mOH mRNA expression by RT-PCR. The level of mOH mRNA was abundant in brain, spinal cord, lung, and uterus, but almost undetectable in spleen, stomach, and smooth muscle (Fig. 6). Among the embryo stages, mOH gene expression was predominantly expressed in day 17, suggesting a development-dependent regulation.

## Discussion

The understanding of estrogen influences on endometrial remodeling in mice was based on the study of immature mice ULF gelatinolytic enzymes. In the present study, we have isolated the large gelatinolytic enzyme complex (~700 kDa) from immature mice ULF using a two-step chromatography plus gelatin zymoglaphy. Detailed characterization of the gelatinolytic enzymes in ULF revealed that all of them belonged to trypsin-like serine proteases, and the 26 kDa gelatinolytic enzyme had the ability to digest type I collagen. This is the first study to demonstrate that the gelatinolytic enzymes form a complex with a novel protein, mOH. mOH was a protease inhibitor specific for these gelatinolytic enzymes, based on two following lines of evidence: First, its primary sequence is highly homologous to that of chicken ovostatin, a well-defined protease inhibitor^34–36^. Second, mOH is specifically associated with gelatinolytic enzymes, as evidenced by the co-elution profile during purification and co-immunoprecipitation (Fig. 4). Although several lines of evidence have implicated both MMPs/TIMPs and PA/inhibitors systems in uterine remodeling processes, our results suggested that the mOH/cognate proteases system might also play a role in uterine remodeling events.

SDS-PAGE analysis under non-reducing conditions indicated the purified gelatinolytic enzyme complex containing a protein with the apparently molecular weight of around 360 kDa. Only mOH protein was identified in ~360 kDa band with the high MASCOT score in mass spectrometry analysis (Table 1). In comparison with the other vertebrate species, the mOH protein sequence shares 82%, 63%, and 45% identity with that of rat A2ml 1, human ovostatin 2 and chicken ovostatin, respectively. Since its primary sequence showed a significant homology with that of chicken ovostatin, the biological function of mOH should be similar to that of chicken ovostatin. The gene expression of chicken ovostatin was up-regulated in the chicken oviduct by estrogen^37^. Its inhibitory mechanism toward proteases is like that of α_2_-MG, and contrasts with many other known mechanisms with the protease-inhibitor complex formation involved in blocking the active site of proteases^36,38^. The reaction of α_2_-MG with a protease needs a proteolytic attack of enzyme on its bait region that can be cleaved by almost all four classes of proteases^39,40^. Reaction of chicken ovostatin with human collagenases resulted in the 165 kDa subunit to be cleaved into 88 and 78 kDa fragments^35^. The similar 88 kDa fragments were also generated by interaction of chicken ovostatin with a variety of proteases^35,38^. However, the effect of chicken ovostatin formed a physiological complex with cognate proteases has not been found. The molecular weight based on the primary sequence of mOH is predicted as 162 kDa. Although the presence of ~ 162 kDa mOH subunit in the purified gelatinolytic enzyme complex was not observed in reducing SDS-PAGE (Fig. 2, lane 4), a protein band with approximately 88 kDa appeared in the gel. Mass spectrometry analysis of the 88 kDa faint band (Fig. 2, lane 4) revealed that this protein was identical to mOH. It suggested that the intact mOH subunit was degraded into 88 kDa protein fragment due to the reaction of mOH with cognate proteases. Indeed, the overwhelming majority of 88 kDa mOH fragment and trace amounts of 162 kDa mOH subunit were observed in immature mice ULF (Fig. 3B, lane 1). This suggested that the inhibitory capacity of mOH to cognate gelatinolytic enzymes in immature mice ULF was close to saturation. The ~360 kDa protein consists of two identical 162 kDa subunits linked by disulfide bridges was supported by detection of trace amounts of 162 kDa mOH subunit in immature mice ULF under reducing condition (Fig. 3B, lane 1). The previous studies have demonstrated that the homologous proteins of mOH such as chicken ovoststin^34^ and human ovostatin 2^41^ were displayed as a single protein band of 165 kDa under reducing conditions. The apparently molecular weight of mOH was estimated to be around 700 kDa by gel filtration on the Sephacryl S-400 column. Under non-reducing conditions, SDS-PAGE analysis indicated that its apparently molecular weight was displayed to around 360 kDa. Taken together, the native mOH molecule is a homotetramer. Two identical subunits were linked by the disulfide bond and two dimers had associated each other without disulfide linkages to form a tetramer.

RT-PCR analysis revealed that mOH is predominantly displayed in brain, spinal cord, lung, and uterus. Of particular note is the presence of mOH gene expression in brain because the 22 kDa gelatinolytic enzyme in this study may correspond to the trypsin-like protease, p22, which previously reported by Sawada *et al*.^42^. They isolated p22 from the incubation medium of rat brain slices and found that the p22 can degrade ECM and stimulating protease-activated receptor-2, suggesting that p22 may involve in neuronal cell death. However, the protein sequence of p22 has not been identified. Although it requires more evidence to verify whether both 22 kDa gelatinolytic enzyme and p22 are the same protein, the biochemical characteristics of 22 kDa gelatinolytic enzyme like p22 include: (1) trypsin-like protease inhibited by benzamidine and (2) the molecular weight in gelatin zymogram. It suggested that mOH might play a role in the inhibition of p22 function in brain. Furthermore, mOH is also prominently expressed in mouse 17 embryonic day. The mOH expression pattern in various embryonic days suggests that it may play a role in embryonic development.

Previous reports indicated that the chicken ovostatin mRNA was expressed in the epithelium of both chicken oviduct and cancerous ovaries of hens^37^. Previous study had also reported that the malignant epithelial cells in ovarian cancer tissues strongly expressed human ovostatin 2 (OVOS2), while the expression of OVOS2 in the adjacent non-cancerous tissues was negative or very weak^41^. Furthermore, up-regulation of OVOS2 gene expression in human melanoma^43^ and ovarian cancer cells^41^ was observed. The levels of OVOS2 protein were increased progressively from benign nevi to melanoma and a high level of OVOS2 protein in melanoma appeared to associate with tumor progression^43^. Recent study showed that the growth speed and weight of the transplanted tumor were significantly suppressed in the BALB/c nude mice subcutaneously injected with OVOS 2 knocked-down A375 melanoma cells^44^. The interaction of tumor cells with ECM through integrins or other cell surface receptors has been suggested to play crucial role in tumor progression^45^. Several cancer cells can stimulate type I collagen synthesis in tumor-associated stromal cells^46–48^. The increased stromal type I collagen expression in mouse mammary tissue significantly increase tumor formation, invasion, and lung metastasis in bi-transgenic mouse model^49^. Several lines of evidence have demonstrated that type I collagen can induce the epithelial-mesenchymal transition, which is characterized by down-regulation of E-cadherin expression observed in various types of cancer cells^50–52^. Our finding suggested that mOH associated with cognate protease may implicate the inhibitory role of type I collagen damage in tumor-stromal interface of these cancers. Previous study also demonstrated that E-cadherin was down-regulated in response to type I collagen in OVOS2-expressed SKOV3 ovarian cancer cells^51^, suggesting that an association between increased OVOS2 protein and the progression of human ovarian cancer was mainly due to the mediator of type I collagen. However, it remained unknown whether inhibition of type I collagen damage is important to cancer progression. Further studies need to be carried out to unravel the effect of mOH/cognate proteases on cancer development.

## Materials and Methods

### Materials

Acrylamide, ammonium persulfate, Tris base, glycine, sodium dodecyl sulfate (SDS), diethylstilbesterol (DES), glutathione (GSH), Triton X-100, gelatin, human placenta type IV collagen, bovine skin type I collagen, mouse albumin, 1-10-phenanthroline, iodoacetamide, pepstatin, bezamidine, chymostatin and leupeptin were purchased from Sigma-Aldrich (St. Louis, MO, USA). Sephacryl S-400, DEAE-Sepharose, glutathione Sepharose 4B, CNBr-activated Sepharose 4B, polyvinylidene difluoride (PVDF) membrane, and pGEX-4T-1 were ordered from GE Healthcare Life Sciences (Piscataway, NJ, USA). The restriction enzymes, *Bam*HI and *Eco*RI, were ordered from New England BioLabs, Inc. (Ipswich, MA, USA). Anti-mouse CLCA3 antibody was purchased from Abcam (Cambridge, UK).

### Ethics statement

The female New Zealand white rabbits were purchased from Livestock Research Institute, Council of Agriculture (Tainan, Taiwan). The care, maintaining and experimental procedures involving rabbits and mice were reviewed and approved by the Institute Animal Care and Use Committee (IACUC) of Fooyin University. Animals were maintained following the institutional guidelines for the care and use of experimental animals.

### Preparation of immature mice ULF

Immature female ICR mice (21 days old) were injected subcutaneously with a daily dose of DES (1 μg) dissolved in corn oil, respectively, for four consecutive days. The mice were sacrificed on day 25 of age. Then, the ULF was collected from mice uterine lumen. The ULF solution containing 2 mM EDTA was centrifuged at 10,000 × *g* for 2 min to remove insoluble components and then stored at −70 °C until further analysis.

### Substrate zymography

The designated samples were mixed with non-reducing sample loading buffer and then subjected to SDS-PAGE analysis with 12% polyacrylamide gels containing gelatin (2 mg/ml), human placenta type IV collagen (1 mg/ml), or bovine skin type I collagen (1 mg/ml). After electrophoresis, the gel slices were washed by the buffer (0.1 M Tris-HCl, pH 7.4, containing 2.5% Triton X-100) two times at room temperature for 18 minutes and then incubated in 0.1 M Tris-HCl (pH 7.4) at 37 °C. After incubation overnight, the gels were stained with Coomassie Brilliant Blue for 20 minutes, followed by destaining in 10% acetic acid solution. The destained gels were scanned by HP Scanjet G3010 photo scanner. Gelatinolytic activities were detected by clear zones indicating the degradation of the substrate and quantified by measuring the area intensity of indicated band subtracted identical background area intensity of same lane by using ImageJ software.

### Protein isolation

1 mL of ULF was fractionated by gel filtration chromatography on the Sephacryl S-400 column (15 mm × 100 cm), developed by PBS buffer containing 2 mM EDTA with a flow rate of 0.2 ml/min at cold room. Each fraction was collected with 3.2 ml. Aliquots (0.3 ml) of the indicated fractions were dialyzed against water, lyophilized, and applied to gelatin zymography for activity assay. The pooled fractions (140 ml) containing 26 kDa gelatinolytic enzyme collected from Sephacryl S-400 chromatography were dialyzed, lyophilized, then dissolved in 20 ml of the equilibrated buffer A (5 mM phophate buffer containing 2 mM EDTA at pH 7.4), and loaded onto the DEAE-Sepharose column (15 mm × 2 cm). After washed with ten volumes of buffer A, the column was developed by a linear gradient from 75 ml of buffer A to 75 ml of buffer A plus 0.4 M NaCl. Each fraction collected 3.6 ml. 0.25 ml withdrawn from the indicated fractions were dialyzed, lyophilized, and then subjected to gelatin zymography for activity assay. Fractions containing 26 kDa gelatinolytic enzyme were pooled, dialyzed against water to remove the excess reagents, lyophilized, and used as an antigen to raise the rabbit anti-mOH antibody.

### Mass spectrometry

The purified gelatinolytic enzyme complex was analyzed by SDS-PAGE and then stained with Coomassie Brilliant Blue. The assigned bands were excised from gel, digested by trypsin, and analyzed by liquid chromatography-electrospray ionization combined with tandem mass spectrometry (LC-MS/MS), respectively.

### Expression and Purification of GST-mOH[870–940] fusion protein

The cDNA of the partial mOH sequence (residues 870–940 amino acids) was amplified from DES-treated immature mice uterus by RT-PCR using the primers (5′-CTC GGA TCC ACT GTA GTG GCT ACA TCC-3′ and 5′-CTC GAA TTC TCA ACC TTC TAC TAC ATT GCT -3′). The resulting product was digested by *Bam*HI/ *Eco*RI and sub-cloned into pGEX 4T-1 to give the vector, pGEX 4T-1-mOH [870–940]. *E. coli* BL21 (DE 3) harboring pGEX-4T-1-mOH[870–940] vector were grown in 1 L Luria-Bertani (LB) broth containing ampicillin (100 μg/ml) at 37 °C. Until the absorbance at 600 nm reached approximately 0.4, the isopropyl β-D-1-thiogalactopyranoside (final concentration of 0.4 mM) was added to induce the protein expression. After four hours, bacteria were harvested by centrifugation at 6,000 × *g* for 15 min. Cells in the pellet were resuspended in the buffer (PBS containing 1 mg/ml lysozyme, 2 mM EDTA, and 1 mM PMSF), incubated for 1 hr at 37 °C, and then ruptured by three freezing and thawing cycles. The lysate was treated with DNase I (1 μg/ml) for 30 min at 37 °C, followed by centrifugation at 6,000 × *g* at 4 °C for 30 min. The supernatant was loaded onto to a column packed with Glutathione Sepharose 4B resin. Unbound proteins were eluded by washing with PBS. The GST-mOH[870–940] fusion protein was eluted with PBS containing 10 mM GSH. The elution was dialyzed against water to remove the excess reagents, lyophilized and stored for preparation of mOH[870–940] antibody. GST-mOH[870–940] antibody recognizes only reduced form of mOH.

### Preparation of purified mOH antibody (POHA)

ULF were purified by gel filtration using a Sephacryl S-400 column (15 mm × 100 cm), developed by PBS. Each fraction collected 3.2 ml. The major proteins of mCLCA3, mOH and C3 in ULF were eluted and collected at fractions 26–30, 32–35 and 38–40, respectively. Fractions containing mCLAC3, mOH and C3 were pooled, respectively, and dialyzed against distilled water and lyophilized. The mOH purified from ULF (15 ml) by using Sephacryl S-400 and DEAE-Sepharose chromatographs was conjugated to 1.0 ml of CNBr-activated Sepharose beads, following the manufacturer’s instruction. This purified mOH also contains C3, albumin and mCLCA3. Therefore, anti-C3, anti-albumin and anti-mCLAC3 antibody in mOH-raised antibodies were removed by absorption with C3-, albumin- and mCLCA3-conjugated Sepharose beads. One milligram each of C3, mCLCA3, and mouse albumin was conjugated to 1.5 ml of CNBr-activated Sepharose beads, following the manufacturer’s instruction. The rabbit anti-mOH antibody (0.5 ml) was applied to affinity chromatography on the column packed with mOH-conjugated beads. Following multiple washes, antibody was eluted with 0.1 M glycine (pH 3), and immediately neutralized by the addition of 1 M Tris-HCl (pH 8). The elution pools (6.0 mL) were subsequently incubated with absorbed beads for 1 hr. The 6.0 mL of purified mOH antibody (POHA) were collected from unbound elution solution and then stored at −70 °C. POHA can recognize the native form of mOH in our assay.

### Western blotting analysis

The designated samples were separated by SDS-PAGE with a linear gradient polyacrylamide gels (4–15%), followed by electroblotting to PVDF membrane, probed with the POHA (250 μl), mOH [870–940] antisera (diluted 1:2000), or mCLCA 3 antibody (diluted 1:2000).

### Co-fractionation analysis

1 mL of ULF was fractionated by Sephacryl S-400 chromatography as described above. Aliquots (0.30 ml) of the indicated fractions (21–47) from gel filtration chromatography were resolved by SDS-PAGE and then applied to Western blotting analysis, probed for non-reduced mOH using POHA or for reduced mCLCA3 using rabbit mCLCA 3 antibodies. In addition, the identical samples were subjected to gelatin zymography for detection of gelatinolytic enzymes.

### Co-immunoprecipitation analysis

An aliquot (60 μl) of protein A-Sepharose beads was incubated with either 5 μg of preimmune antibody (PIA) or 5 ml of POHA, rotated at room temperature for one hour and then was washed with 0.1 ml PBS for three times. All components were added with 30 μl of ULF and then rotated at room temperature for one hour. The beads were washed with 0.1 ml PBS for three times, followed by centrifugation. The precipitated proteins were re-suspended with two volumes of SDS gel loading buffer (30 μl). Then, aliquots (6 μl) of immunoprecipitated proteins were subjected to gelatin zymography.

### Inhibition assay

Aliquot (0.3 mL) of the gelatinolytic enzymes complex isolated from Sephacryl S-400 chromatography were analyzed by gelatin zymogrphy in the presence of different protein inhibitors, including 1–10-phenanthroline (1 mM), iodoacetamide (0.1 mM), pepstatin (20 μM), bezamidine (2 mM), chymostatin (100 μM) or leupeptin (100 μM).

### Determination of optimal pH for gelatinolytic enzyme activity

The partially purified gelatinolytic enzymes complex obtained from Sephacryl S-400 chromatography was subjected to gelatin zymography. Each lane of gel was excised and washed by the buffer (0.1 M Tris-HCl, pH 7.4, containing 2.5% Triton X-100) two times at room temperature for 18 minutes and then incubated in buffers with different pH values, respectively. The buffers included 0.1 M citrate (pH 5.0 and 6.0), 0.1 M Tris (7.4 and 8.0) or 0.1 M glycine (pH 9.0, 10.0 and 10.5). After incubation at 37 °C overnight, the sliced gels were stained with Coomassie Brilliant Blue and then destained by 10% acetic acid.

### Type I collagen degradation assay

The 6 μg of mOH-proteases complex from purification was separated by 12% polyacrylamide gel with SDS under non-reducing condition. After electrophoresis, the gel was washed by the buffer (0.1 M Tris-HCl, pH 7.4, and containing 2.5% Triton X-100) at room temperature for 15 minutes three times to remove SDS. The protein bands with the apparent molecular weights of 26 and of 23/22 kDa, respectively, were excised, ground, and rotationally incubated with 100 μL of type I collagen (1 mg/mL). After overnight incubation at 37 °C, aliquots (8 μL) of the supernatant were subjected to analysis by SDS-PAGE using a linear gradient polyacrylamide gel (4–15%).

### Reverse transcription – polymerase chain reaction (RT-PCR)

The RT-PCR was used to determine the tissue distribution of mOH mRNA transcripts in mice. The multi-tissue mRNA panel of mouse was obtained from Clontech Laboratories, Inc. (Mountain View, CA, USA). Reverse transcription reaction followed the manufacturer’s instruction (Invitrogen, Grand Island, NY, USA). The resulting product was served as a template for PCR amplification of the mOH transcript by the primers (5′-GCT ACA CTG GAG CTC GTG AAA GC-3′ and 5′-GGT GCT TGA TCA GGC TCT TGG TAG-3′). GAPDH was amplified in parallel by the primers (5′-ACC ACA GTC CAT GCC ATC AC-3′ and 5′-TCC ACC ACC CTG TTG CTG TA-3′) and acted as an internal control.

## Supplementary information

Supplementary Information

## Data Availability

All data generated or analyzed during this study are included in this published article (and its supplementary information files).
