# Supplementary material for: A complex of novel protease inhibitor, ovostatin homolog, with its cognate proteases in immature mice uterine luminal fluid
Source: Sci Rep. 2019 Mar 21;9:4973. doi: 10.1038/s41598-019-41426-4 (PMC6428836; doi:10.1038/s41598-019-41426-4)
Supplement: Supplementary file 1 — Supplementary Information [file 41598_2019_41426_MOESM1_ESM.pdf]

Supplementary Information for:

A complex of novel protease inhibitor, ovostatin homolog, with its cognate proteases in immature mice uterine luminal fluid

Hsien-Lu Huang[a,1,*], Szu-Chin Li[b,c,d,1],and Jin-Fong Wu[a]

[a]Department of Nutrition and Health Science, Fooyin University, Kaohsiung City 83102, Taiwan.
[b]Department of Biomedical Sciences, National Chung Cheng University, Chiayi 62102, Taiwan.
[c]School of Medicine, Tzu Chi University, Hualian 97004, Taiwan
[d]Division of Hematology-Oncology, Department of Internal medicine, Dalin Tzu Chi Hospital, Buddhist Tzu Chi Medical Foundation, Chiayi 62247, Taiwan

Corresponding author:
Hsien-Lu Huang: mt103@fy.edu.tw

Supplementary Figure S1

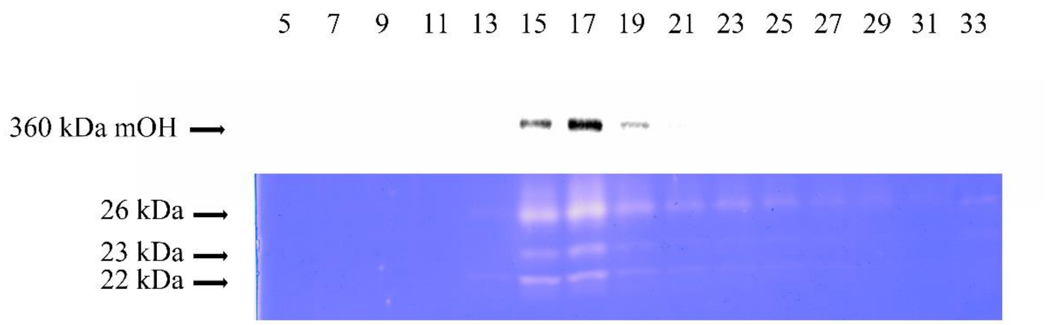

Fig. S1. Co-fractionation of mOH with gelatinolytic enzymes during purification by DEAE-Sepharose chromatography. The pooled fractions from gel filtration were subjected to DEAE-Sepharose chromatography as described in Materials and Methods. Aliquots (0.25 ml) of the indicated fractions (5-33) from DEAE-Sepharose chromatogrphy were subjected to Western blotting (upper panel), probed for mOH using POHA and to gelatin zymography (lower panel) for detection of gelatinolytic enzymes, respectively. The original immunoblot and scan of gelatin zymogram are shown in Supplementary Fig. S4.

Supplementary Fig. S2.1: The protein view of band 1 from MASCOT search results.

MASCOT Search Results

Protein View: NP_001001179.2

ovostatin homolog precursor [Mus musculus]

**Database:**	NCBIprot
**Score:**	1120
**Monoisotopic mass (M_r):**	162238

Protein sequence coverage: 15%

Matched peptides shown in ***bold red***.

1 MVPTILLSAL LLHFTDVVAA EPR**YILWVSS** **VVQR**FSSEKA CLHLLNLNES

51 VSLSVTLEYD GSSTTIFDQP VDEGNFYACA DFKVSQMSSE QLAFVALLVQ

101 GNTLKISERR SVAIAAEENA TFVQTDTPVH KPGDTVHFRV VTLNIWLKPV

151 DDLYPLITVQ DPQSNVIFQW INVTTFRNIT QLSFQLTPEP ILGDYTIVIK

201 TQSGTTVMDH FTVNRDVLPK FEVELTAPET ITIADSQFQM VTCAK**YTYGQ**

251 **PVQGK**AQIKV CRELFSPAHC ESNENEICEQ FTVQLK**DGCA** **SHIINTK**VFQ

301 LDRSGLFMTL NVNEVVTESG TGVQMSKTHS VFITSVLGTV SFENMDPFYR

351 RGITYFGTLK **FSGPNNTPLV** **DK**LLQLELDG KPVGNYTTDE NGEARFSINT

401 SEIFGAQISL KAVYVRPRSC HRSSWLSPEY LDAYFSASR**F** **YSQTSSFTK**I

451 ILEPKQLPCD QEK**MFSVLYS** **LNPEAYK**EAS DVTFFYLVMV RGGISRSGQK

501 QVRVQAWNGN FSFPISINAD LAPSADLFVY TLHPSGEIVA DNVRLQIEKC

551 FKNK**VSINFS** **R**DKDLPGSNT SVHLQAAPDS FCALRAVDKS ALLLNHGQEM

601 TPESVYFTLP YIHQYGYFYN GLNLDDQQAE PCIPQKDLFY NGLYYTPTGN

651 IWDGDLSNLL SNMGLK**IFTN** **LHYRKPEVCS** **SQENQPLLR**T FDHPNERIMM

701 YGGGAPPSSA FHDSVDSISH AKVAIKETVR TNFPRTWIWN LVSVDSSGTA

751 NVSFLVPDTI TQWEASAFCV NGNAGFGISP KVSLQISQPF FVEVTSPFSV

801 VRSEQSDMVV TVFNYLTTCV EISVQLEASE NYEASINTQR **NTDSEVLQAG**

851 **EQK**TYVWTII PKTLGKVNVT VVATSKQSRA CPNDASKEQD VHWKDTVVK**T**

901 **MLVEAEGIEK** **EATQSFLICP** **K**GTKASK**QTL** **LELPSNVVEG** **SVR**SFVTIVG

951 DILGVAMQNL ESLLQMPYGC GEQNIAQLAS DVYILDYLK**A** **TDQLTEELK**S

1001 KAQRLLSNGY QNHLSFKNYD GSYDVFCQSN QEGSTWLSAL SFKTVEKMKE

1051 YIFIEETVPK **QTLIWLVK**KQ KSNGCFRRDE KHVDTAQEGR EGDQEDIALT

1101 AYVVGVFLEV GLNASFPALR NGLYCLEEAF SNGVTNGYTQ AILAYVFALA

1151 GKEQQAKSLL SILDKSATK**T** **NNMIYWER**DE KPETDNSPSF IPSALSGETE

1201 KTCYVLLAVL SQDTQDLDYA SK**IVQWLAQR** MNSHGGFSAM QDTTVCLLAL

1251 TQYMKLTGSN PQNTITLSSE ESEEVFYVNR NKRLLVQHSK VSKGHQQYTV

1301 DVEGDGCSFI QATLR**YNVPL** **PKEASGFSLS** **VK**TGKSNSSD EFQTKFELTV

1351 TLTYTGARES SVTVLVDVK**M** **LSGFTPVVSS** **TEELK**FNSQV TKTDIK**NGHV**

1401 **LFYLENVPK**E ATSLTFSIEQ TNHVANIQPA PVTVYSYEKG EYAFDSYNIN

1451 SISDSQ

Supplementary Fig. S2.2: The protein view of band 2 from MASCOT search results.

MASCOT Search Results

Protein View: NP_033908.2

complement C3 preproprotein [Mus musculus]

**Database:**	NCBIprot
**Score:**	1223
**Monoisotopic mass (M_r):**	186366
**Calculated pI:**	6.29

Protein sequence coverage: 38%

Matched peptides shown in ***bold red***.

**1**	MGPASGSQLL	VLLLLLASSP	LALGIPMYSI	ITPNVLRLES	EETIVLEAHD
**51**	AQGDIPVTVT	VQDFLKRQVL	TSEK**TVLTGA**	**SGHLR**SVSIK	IPASKEFNSD
**101**	KEGHK**YVTVV**	**ANFGETVVEK**	AVMVSFQSGY	LFIQTDK**TIY**	**TPGSTVLYRI**
**151**	**FTVDNNLLPV**	**GKTVVILIET**	**PDGIPVK**RDI	LSSNNQHGIL	PLSWNIPELV
**201**	NMGQWKIR**AF**	**YEHAPKQIFS**	**AEFEVKEYVL**	**PSFEVR**VEPT	ETFYYIDDPN
**251**	GLEVSIIAKF	LYGK**NVDGTA**	**FVIFGVQDGD**	**KKISLAHSLT**	**RVVIEDGVGD**
**301**	**AVLTRKVLME**	**GVRPSNADAL**	**VGK**SLYVSVT	VILHSGSDMV	EAER**SGIPIV**
**351**	**TSPYQIHFTK**	TPK**FFKPAMP**	**FDLMVFVTNP**	**DGSPASKVLV**	**VTQGSNAK**AL
**401**	TQDDGVAKLS	INTPNSRQPL	TITVRTK**KDT**	**LPESR**QATK**T**	**MEAHPYSTMH**
**451**	**NSNNYLHLSV**	**SRMELKPGDN**	**LNVNFHLRTD**	**PGHEAK**IR**YY**	**TYLVMNK**GKL
**501**	LKAGRQVREP	GQDLVVLSLP	ITPEFIPSFR	**LVAYYTLIGA**	**SGQREVVADS**
**551**	**VWVDVKDSCI**	**GTLVVK**GDPR	**DNHLAPGQQT**	**TLR**IEGNQGA	RVGLVAVDKG
**601**	VFVLNKKNKL	TQSKIWDVVE	K**ADIGCTPGS**	**GKNYAGVFMD**	**AGLAFKTSQG**
**651**	**LQTEQRADLE**	**CTKPAAR**RRR	SVQLMERRMD	KAGQYTDKGL	RKCCEDGMRD
**701**	IPMRYSCQRR	ARLITQGENC	IKAFIDCCNH	ITKLREQHRR	DHVLGLAR**SE**
**751**	**LEEDIIPEED**	**IISR**SHFPQS	WLWTIEELKE	PEKNGISTKV	MNIFLKDSIT
**801**	TWEILAVSLS	DKKGICVADP	YEIR**VMQDFF**	**IDLR**LPYSVV	RNEQVEIRAV
**851**	LFNYREQEEL	KVR**VELLHNP**	**AFCSMATAK**N	RYFQTIKIPP	K**SSVAVPYVI**

901 **VPLKIGQQEV** **EVKAAVFNHF** **ISDGVK**KTLK VVPEGMRINK **TVAIHTLDPE**

951 **K**LGQGGVQKV DVPAADLSDQ VPDTDSETRI ILQGSPVVQM AEDAVDGERL

1001 KHLIVTPAGC GEQNMIGMTP TVIAVHYLDQ TEQWEKFGIE KRQEALELIK

1051 **KGYTQQLAFK** QPSSAYAAFN NRPPSTWLTA YVVKVFSLAA NLIAIDSHVL

1101 CGAVKWLILE KQKPDGVFQE DGPVIHQEMI GGFRNAKEAD VSLTAFVLIA

1151 LQEARDICEG QVNSLPGSIN KAGEYIEASY MNLQRPYTVA IAGYALALMN

1201 KLEEPYLGKF LNTAKDRNRW EEPDQQLYNV EATSYALLAL LLLKDFDSVP

1251 PVVRWLNEQR YYGGGYGSTQ ATFMVFQALA QYQTDVPDHK DLNMDVSFHL

1301 PSRSSATTFR LLWENGNLLR SEETK**QNEAF** **SLTAK**GKGR**G** **TLSVVAVYHA**

1351 **K**LKSKVTCKK FDLR**VSIRPA** **PETAK**KPEEA K**NTMFLEICT** **K**YLGDVDATM

1401 SILDISMMTG FAPDTK**DLEL** **LASGVDR**YIS KYEMNKAFSN K**NTLIIYLEK**

1451 **ISHTEEDCLT** **FKVHQYFNVG** **LIQPGSVKVY** **SYYNLEESCT** **RFYHPEKDDG**

1501 **MLSKLCHSEM** **CRCAEENCFM** **QQSQEK**INLN VRLDK**ACEPG** **VDYVYK**TELT

1551 NIELLDDFDE YTMTIQQVIK **SGSDEVQAGQ** **QR**KFISHIKC RNALKLQKGK

1601 KYLMWGLSSD LWGEKPNTSY IIGK**DTWVEH** **WPEAEECQDQ** **K**YQKQCEELG

1651 AFTESMVVYG CPN

Supplementary Fig. S2.3: The protein view of band 3 from MASCOT search results.

MASCOT Search Results

Protein View: AAH28343.1

Chloride channel calcium activated 3 [Mus musculus]

**Database:**	NCBIprot
**Score:**	625
**Monoisotopic mass (M_r):**	100043
**Calculated pI:**	5.67

Protein sequence coverage: 13%

Matched peptides shown in ***bold red***.

1 MESLKSPVFL LILHLLEGVL SESLIQLNNN GYEGIVIAID HDVPEDEALI

51 QHIK**DMVTQA** **SPYLFEATGK** RFYFK**NVAIL** **IPESWK**AKPE YTRPKLETFK

101 NADVLVSTTS PLGNDEPYTE HIGACGEKGI RIHLTPDFLA GK**KLTQYGPQ**

151 **DR**TFVHEWAH FRWGVFNEYN NDEKFYLSKG KPQAVRCSAA ITGKNQVRRC

201 QGGSCITNGK CVIDRVTGLY KDNCVFVPDP HQNEKASIMF NQNINSVVEF

251 CTEKNHNQEA PNDQNQRCNL RSTWEVIQES EDFKQTTPMT AQPPAPTFSL

301 LQIGQR**IVCL** **VLDK**SGSMLN DDRLNRMNQA SRLFLLQTVE QGSWVGMVTF

351 DSAAYVQSEL KQLNSGADRD LLIKHLPTVS AGGTSICSGL RTAFTVIKKK

401 YPTDGSEIVL LTDGEDNTIS SCFDLVK**QSG** **AIIHTVALGP** **AAAK**ELEQLS

451 KMTGGLQTYS SDQVQNNGFV DAFAALSSGN AAIAQHSIQL ESRGVNLQNN

501 QWMNGSVIVD SSVGKDTLFL ITWTTHPPTI FIWDPSGVEQ NGFILDTTTK

551 **VAYLQVPGTA** **K**VGFWKYSIQ ASSQTLTLTV TSRAASATLP PITVTPVVNK

601 NTGKFPSPVT VYASIRQGAS PILR**ASVTAL** **IESVNGKTVT** **LELLDNGAGA**

651 **DATK**NDGVYS R**FFTAFDANG** **R**YSVK**IWALG** **GVTSDR**QRAA PPKNRAMYID

701 GWIEDGEVRM NPPRPETSYV QDKQLCFSRT SSGGSFVATN VPAAAPIPDL

751 FPPCQITDLK ASIQGQNLVN LTWTAPGDDY DHGRASNYII RMSTSIVDLR

801 DHFNTSLQVN TTGLIPKEAS SEEIFEFELG GNTFGNGTDI FIAIQAVDKS

851 NLKSEISNIA RVSVFIPAQE PPIPEDSTPP CPDISINSTI PGIHVLKIMW

901 KWLGEMQVTL GLH

Supplementary Fig. S2.4: The protein view of band 4 from MASCOT search results.

MASCOT Search Results

Protein View: NP_033784.2

serum albumin preproprotein [Mus musculus]

**Database:**	NCBIprot
**Score:**	762
**Monoisotopic mass (M_r):**	68648
**Calculated pI:**	5.75

Protein sequence coverage: 23%

Matched peptides shown in ***bold red***.

**1**	MKWVTFLLLL	FVSGSAFSRG	VFRREAHKSE	IAHR**YNDLGE**	**QHFKGLVLIA**
**51**	**FSQYLQK**CSY	DEHAK**LVQEV**	**TDFAKTCVAD**	**ESAANCDK**SL	HTLFGDKLCA
**101**	IPNLRENYGE	LADCCTKQEP	ERNECFLQHK	DDNPSLPPFE	RPEAEAMCTS
**151**	FKENPTTFMG	HYLHEVARRH	PYFYAPELLY	YAEQYNEILT	QCCAEADKES
**201**	CLTPKLDGVK	EKALVSSVRQ	RMKCSSMQKF	GERAFKAWAV	ARLSQTFPNA
**251**	DFAEITKLAT	DLTKVNKECC	HGDLLECADD	RAELAK**YMCE**	**NQATISSK**LQ
**301**	TCCDKPLLKK	AHCLSEVEHD	TMPADLPAIA	ADFVEDQEVC	KNYAEAKDVF
**351**	LGTFLYEYSR	RHPDYSVSLL	LRLAKKYEAT	LEKCCAEANP	PACYGTVLAE
**401**	FQPLVEEPKN	LVK**TNCDLYE**	**KLGEYGFQNA**	**ILVR**YTQKAP	QVSTPTLVEA
**451**	ARNLGRVGTK	CCTLPEDQR**L**	**PCVEDYLSAI**	**LNR**VCLLHEK	**TPVSEHVTK**C
**501**	CSGSLVERRP	CFSALTVDET	YVPKEFKAET	FTFHSDICTL	PEKEKQIKK**Q**
**551**	**TALAELVK**HK	PKATAEQLK**T**	**VMDDFAQFLD**	**TCCKAADKDT**	**CFSTEGPNLV**
**601**	**TR**CKDALA				

Supplementary Fig. S2.5: The protein view of band 5 from MASCOT search results.

MASCOT Search Results

Protein View: NP_001001179.2

ovostatin homolog precursor [Mus musculus]

**Database:**	NCBIprot
**Score:**	303
**Monoisotopic mass (M_r):**	162238
**Calculated pI:**	5.14

Protein sequence coverage: 9%

Matched peptides shown in ***bold red***.

1 MVPTILLSAL LLHFTDVVAA EPR**YILWVSS** **VVQR**FSSEKA CLHLLNLNES

51 VSLSVTLEYD GSSTTIFDQP VDEGNFYACA DFKVSQMSSE QLAFVALLVQ

101 GNTLKISERR SVAIAAEENA TFVQTDTPVH KPGDTVHFRV VTLNIWLKPV

151 DDLYPLITVQ DPQSNVIFQW INVTTFRNIT QLSFQLTPEP ILGDYTIVIK

201 TQSGTTVMDH FTVNRDVLPK FEVELTAPET ITIADSQFQM VTCAK**YTYGQ**

251 **PVQGK**AQIKV CRELFSPAHC ESNENEICEQ FTVQLK**DGCA** **SHIINTK**VFQ

301 LDRSGLFMTL NVNEVVTESG TGVQMSKTHS VFITSVLGTV SFENMDPFYR

351 RGITYFGTLK **FSGPNNTPLV** **DK**LLQLELDG KPVGNYTTDE NGEARFSINT

401 SEIFGAQISL KAVYVRPRSC HRSSWLSPEY LDAYFSASR**F** **YSQTSSFTK**I

451 ILEPKQLPCD QEKMFSVLYS LNPEAYKEAS DVTFFYLVMV RGGISRSGQK

501 QVRVQAWNGN FSFPISINAD LAPSADLFVY TLHPSGEIVA DNVRLQIEKC

551 FKNKVSINFS RDKDLPGSNT SVHLQAAPDS FCALRAVDKS ALLLNHGQEM

601 TPESVYFTLP YIHQYGYFYN GLNLDDQQAE PCIPQKDLFY NGLYYTPTGN

651 IWDGDLSNLL SNMGLK**IFTN** **LHYR**KPEVCS SQENQPLLRT FDHPNERIMM

701 YGGGAPPSSA FHDSVDSISH AKVAIKETVR TNFPRTWIWN LVSVDSSGTA

751 NVSFLVPDTI TQWEASAFCV NGNAGFGISP KVSLQISQPF FVEVTSPFSV

801 VRSEQSDMVV TVFNYLTTCV EISVQLEASE NYEASINTQR **NTDSEVLQAG**

851 **EQK**TYVWTII PKTLGKVNVT VVATSKQSRA CPNDASKEQD VHWKDTVVK**T**

901 **MLVEAEGIEK EATQSFLICP K**GTKASKQTL LELPSNVVEG SVRSFVTIVG

951 DILGVAMQNL ESLLQMPYGC GEQNIAQLAS DVYILDYLK**A TDQLTEELK**S

1001 KAQRLLSNGY QNHLSFKNYD GSYDVFCQSN QEGSTWLSAL SFKTVEKMKE

1051 YIFIEETVPK QTLIWLVKKQ KSNGCFRRDE KHVDTAQEGR EGDQEDIALT

1101 AYVVGVFLEV GLNASFPALR NGLYCLEEAF SNGVTNGYTQ AILAYVFALA

1151 GKEQQAKSLL SILDKSATKT NNMIYWERDE KPETDNSPSF IPSALSGETE

1201 KTCYVLLAVL SQDTQDLDYA SK**IVQWLAQR** MNSHGGFSAM QDTTVCLLAL

1251 TQYMKLTGSN PQNTITLSSE ESEEVFYVNR NKRLLVQHSK VSKGHQQYTV

1301 DVEGDGCSFI QATLR**YNVPL PK**EASGFSLS VKTGKSNSSD EFQTKFELTV

1351 TLTYTGAR**ES SVTVLVDVK**M LSGFTPVVSS TEELKFNSQV TKTDIKNGHV

1401 LFYLENVPKE ATSLTFSIEQ TNHVANIQPA PVTVYSYEKG EYAFDSYNIN

1451 SISDSQ

Supplementary Figure S3

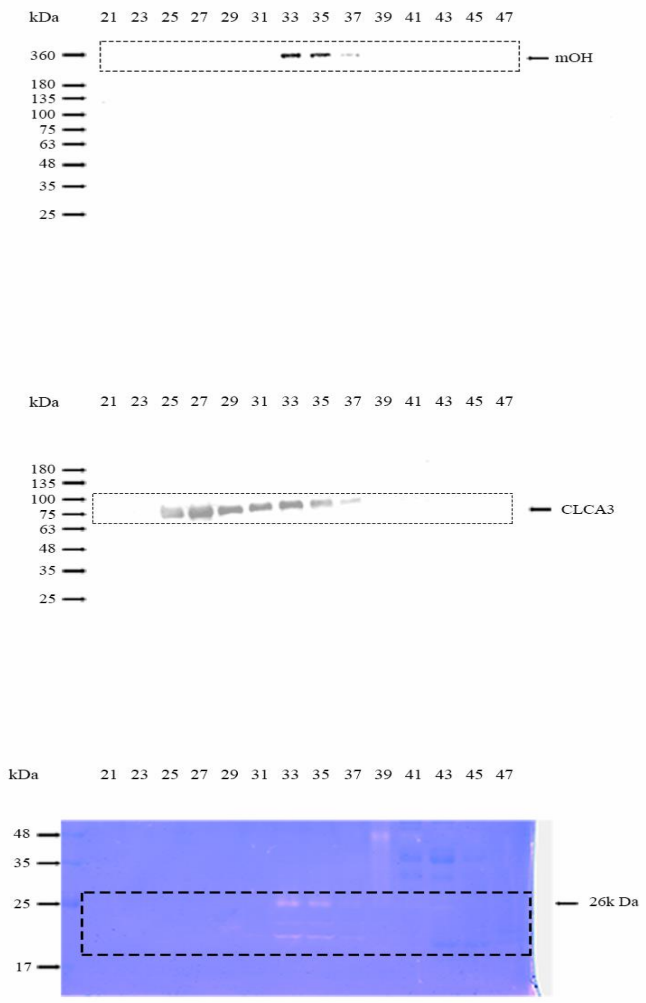

Fig. S3. The original immunoblots and gelatin zymogram of cropped images shown in Fig 4A. Western blot analysis was performed and probed for mOH (upper panel) or mouse CLCA3 (middle panel). The gelatinolytic enzyme with an apparent molecular weight of 26 kDa was observed in gelatin zymogram (lower panel).

Supplementary Figure S4

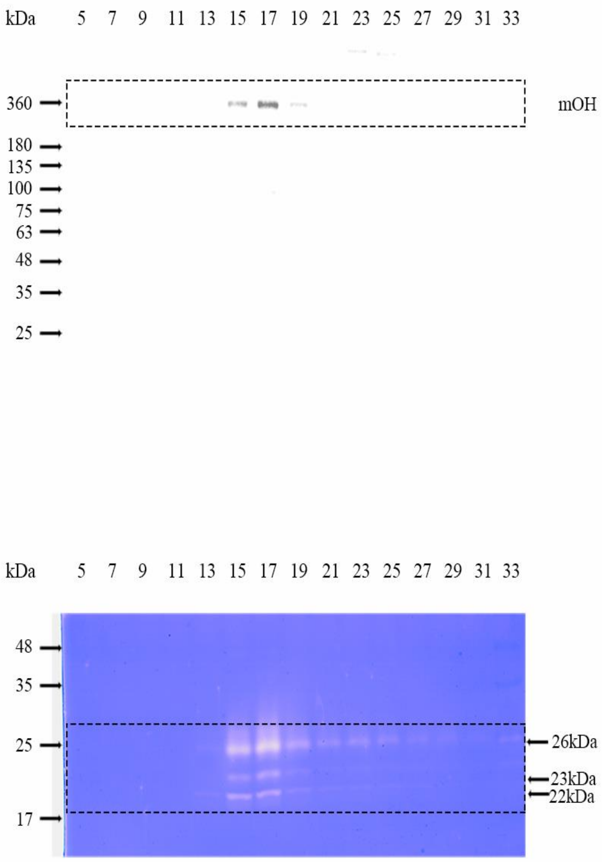

Fig. S4. The original immunoblot and gelatin zymogram of cropped images shown in Fig. S1.

Supplementary Figure S5

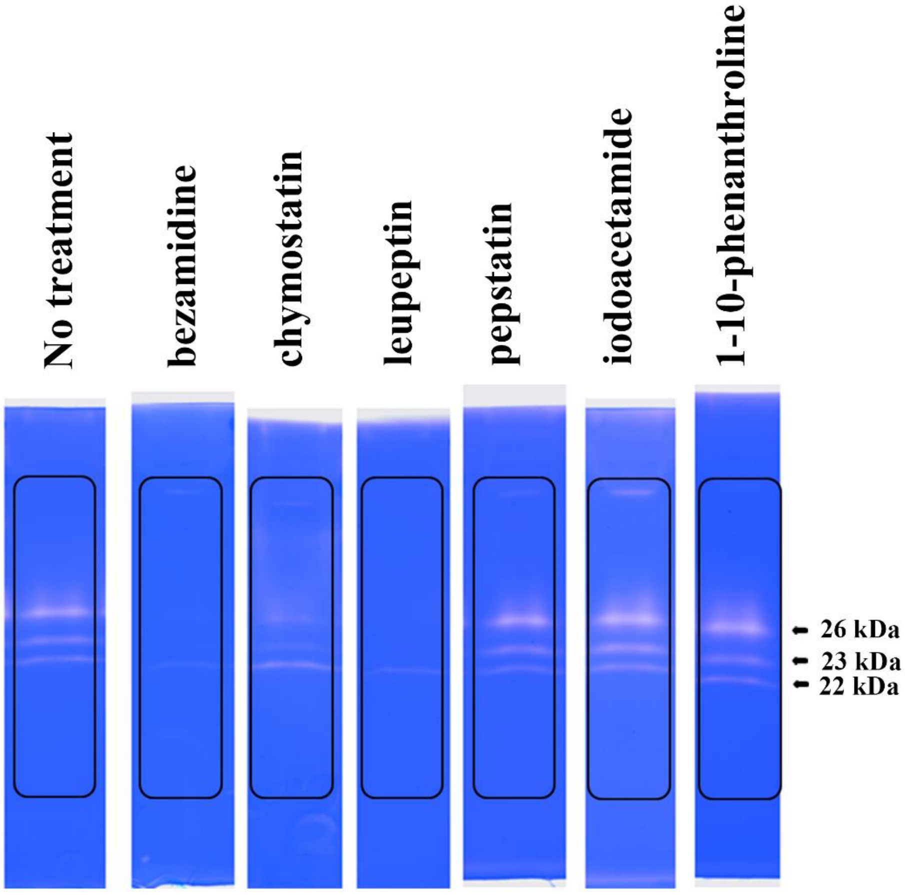

Fig. S5. Original gelatin zymograms of cropped images shown in Fig. 5A.

Supplementary Figure S6

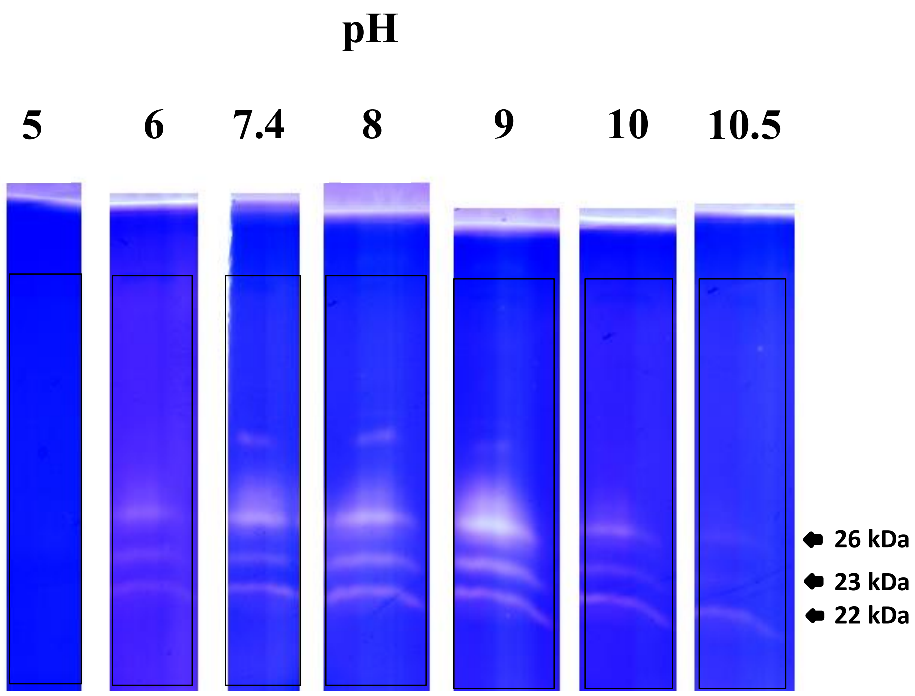

Fig. S6. Original gelatin zymograms of cropped images shown in Fig. 5B.

Supplementary Figure S7

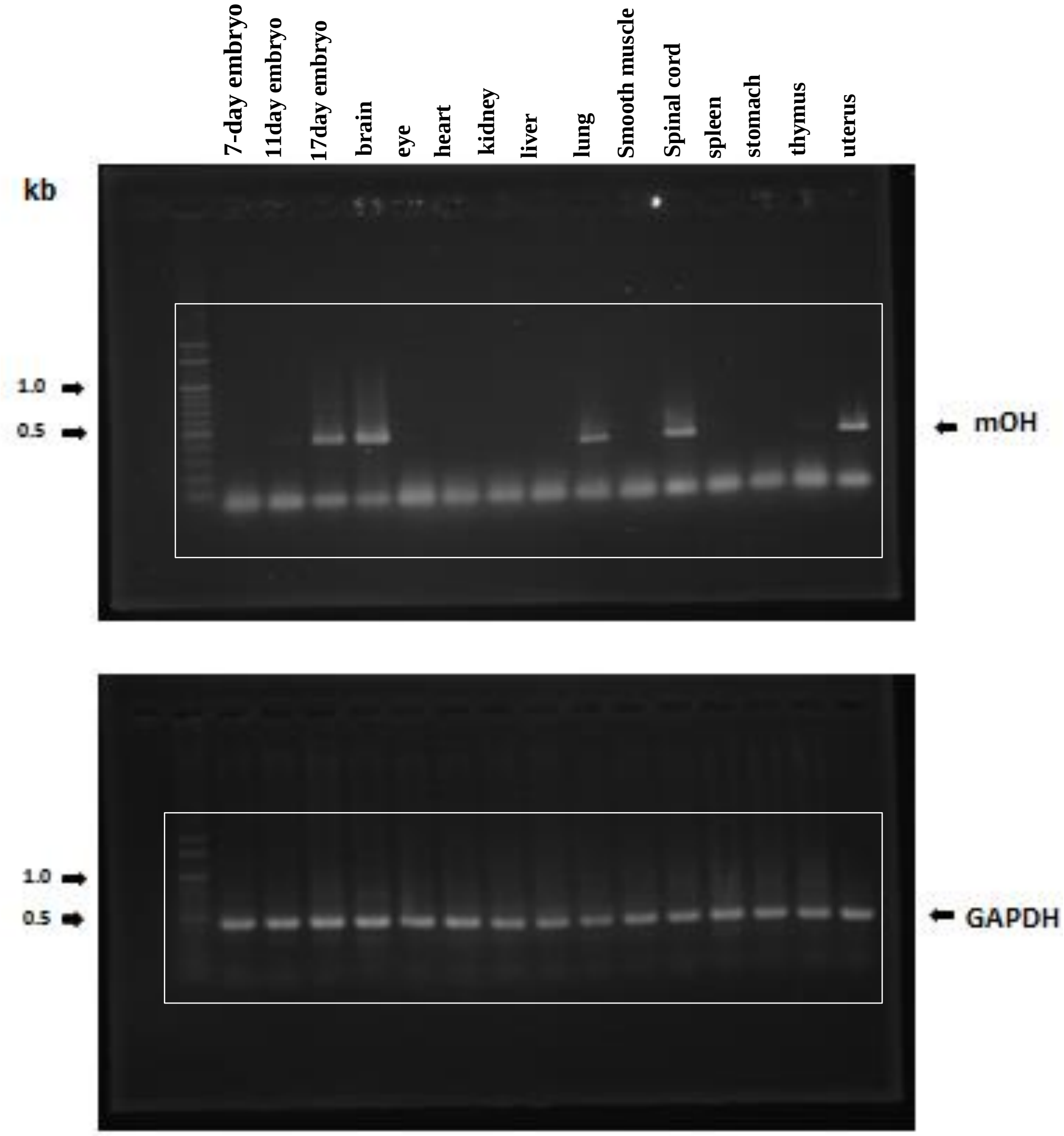

Fig. S7. Agarose gels of cropped images shown in Fig 6.
